# Development of a neuro-fuzzy technique for automated parameter optimization of inverse treatment planning

**DOI:** 10.1186/1748-717X-4-39

**Published:** 2009-09-25

**Authors:** Florian Stieler, Hui Yan, Frank Lohr, Frederik Wenz, Fang-Fang Yin

**Affiliations:** 1 Department of Radiation Oncology, University Medical Center Mannheim, University of Heidelberg, 68167 Mannheim, Germany; 2 Department of Radiation Oncology, Duke University Medical Center, Durham, NC 27710, USA

## Abstract

**Background:**

Parameter optimization in the process of inverse treatment planning for intensity modulated radiation therapy (IMRT) is mainly conducted by human planners in order to create a plan with the desired dose distribution. To automate this tedious process, an artificial intelligence (AI) guided system was developed and examined.

**Methods:**

The AI system can automatically accomplish the optimization process based on prior knowledge operated by several fuzzy inference systems (FIS). Prior knowledge, which was collected from human planners during their routine trial-and-error process of inverse planning, has first to be "translated" to a set of "if-then rules" for driving the FISs. To minimize subjective error which could be costly during this knowledge acquisition process, it is necessary to find a quantitative method to automatically accomplish this task. A well-developed machine learning technique, based on an adaptive neuro fuzzy inference system (ANFIS), was introduced in this study. Based on this approach, prior knowledge of a fuzzy inference system can be quickly collected from observation data (clinically used constraints). The learning capability and the accuracy of such a system were analyzed by generating multiple FIS from data collected from an AI system with known settings and rules.

**Results:**

Multiple analyses showed good agreements of FIS and ANFIS according to rules (error of the output values of ANFIS based on the training data from FIS of 7.77 ± 0.02%) and membership functions (3.9%), thus suggesting that the "behavior" of an FIS can be propagated to another, based on this process. The initial experimental results on a clinical case showed that ANFIS is an effective way to build FIS from practical data, and analysis of ANFIS and FIS with clinical cases showed good planning results provided by ANFIS. OAR volumes encompassed by characteristic percentages of isodoses were reduced by a mean of between 0 and 28%.

**Conclusion:**

The study demonstrated a feasible way to automatically perform parameter optimization of inverse treatment planning under guidance of prior knowledge without human intervention other than providing a set of constraints that have proven clinically useful in a given setting.

## Introduction

Inverse treatment planning has been widely used in the optimization of intensity-modulated radiation therapy (IMRT) to achieve the desired dose distribution by balancing the priorities between planning target and critical organs [1]. Bortfeld et. al. [2] discussed multiple treatment techniques to reduce the delivery time. A remaining goal is to reduce the planning time by automating parts of the planning process. Most current IMRT treatment planning systems provide an interactive user interface to optimize the IMRT plan by editing the dose-volume points and priority weights for each anatomical structure, such as planning target volume (PTV) and organs at risk (OAR) online, an approach commonly named "constraint based optimization". The purpose of the inverse planning optimization is to find the solution in this defined space in order to minimize the values of the objective function. If the minimum value is found in this defined space, the achieved dose distribution is optimal. In brief, the parameter optimization of inverse planning consists of three steps: (1) determine the candidate values for those parameters (constraints and priorities) making up an objective function which is done by human planners; (2) resolve the objective function; and (3) evaluate the resulting dose plan according to certain criteria. These three steps are performed sequentially and repetitively until an optimal solution is found. Based on own experiences and judging from the published experience of others [3,4] conventional constraint based optimization often needs adjustments of constraints in an iterative fashion for most new cases and is therefore time consuming. The reasons for this need for interactivity are both technical and clinical. On a technical level, based on their values, 2D intensity maps can be generated using one type of general optimization algorithms (deterministic and stochastic approaches). Due to certain limitations of the optimization algorithms, frequently a sub-optimal solution is achieved. Limitations for IMRT optimization algorithms are, among others, that negativity of the intensity map is not allowed, that the capability of the planning system find/reach global extrema is limited, that local extrema "trap" the system, that optimization is often performed regarding fluence and not taking into account limitations of segmentation at an earlier stage in the optimization process, etc. On a clinical level, an optimal solution, however, is not only defined by a minimum of the cost function but it has to be related with the individual clinical case and many parameters not included in the cost function itself. This explains why, in addition to improve the processing of the cost function, inserting "human knowledge" into the process may further shorten the hands-on time during treatment planning.

Substantial effort was made to automate this process under the guidance of human knowledge. Li and Yin introduced the fuzzy logic theory in converting the linguistic expression of human knowledge into the trading-off procedure of parameter optimization in inverse planning [5]. They demonstrated that human knowledge can be properly handled by fuzzy logic and applied to inverse treatment planning. Later they employed an 'original' fuzzy inference system (oFIS) to simulate the parameter optimization procedure of inverse planning to replace the routine procedure performed by a human planner [6,7]. Most recently, this AI approach was implemented in a clinical treatment planning system. Based on this platform several clinical cases were examined, which indicated that the dose plans achieved by the AI-approach were comparable or improved over those achieved by human planners in most of the tested cases [8].

The model parameters of the fuzzy inference system still had to be determined manually by a single human expert in a trial-and-error manner based on clinical knowledge ("rules" have to be created directly) and represent knowledge of one single planner only. To make this model selection procedure convenient for clinical use, a learning technique based on neuro-fuzzy systems originally proposed for intelligence control was used for the current study. Based on this approach, a fuzzy inference system can be automatically built from practical data ('rules' are created by a neuro-fuzzy function approximation system, based on "constraints" as usually used in an inverse treatment planning process) without further human intervention.

The neural-fuzzy system "Neuro-Fuzzy Function Approximation (NEFPROX)" in the open source software NFIDENT [9-12] used for this study is briefly introduced. We report the results of this study to evaluate the learning capability of this technique by comparing oFIS with our adaptive neuro fuzzy inference system (ANFIS, trained by oFIS) on a system-level by analyzing rules and on an operational level on one single clinical case. In a second step, we analyzed multiple clinical examples, optimized with ANFIS which was then built and trained by human knowledge and embedded into a commercial treatment planning system (TPS).

The purpose of the study was to establish and evaluate a system that reduces the amount of interaction between a human planner and an inverse treatment planning system during the iterative process of generating inverse treatment plans.

## Materials and methods

### Introduction of the fuzzy inference system (FIS) concept

**Figure 1:**
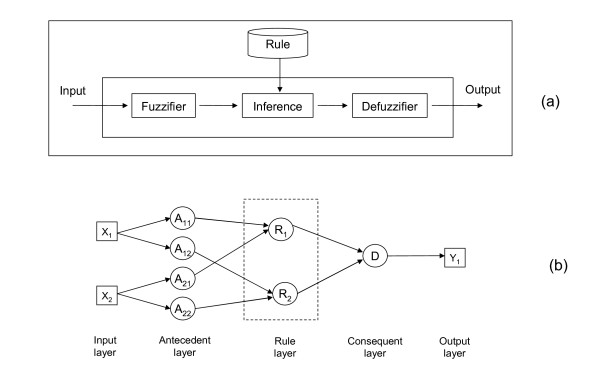
**Fuzzy inference systems**. (a) A Mamdani-type FIS and (b) a fuzzy inference system as neural network.

In 1965, Zadeh proposed a new approach to characterize non-probabilistic uncertainties which is called fuzzy sets [13-16]. This concept found various industrial applications including automatic control, signal processing and decision-making, to name a few. In simulating the reasoning process which is generally conducted by a human, the fuzzy inference system (FIS) was developed by Mamdani which was later implemented in various industrial applications [17-19]. A Mamdani-type FIS consists of three components: fuzzifier, inference engine, and defuzzifier as shown in figure 1a.

The fuzzifier processes the inputs according to the membership function for the inputs. The inference part handles the resulting values and according to the rule base the consequences are computed. The consequences are then converted to the final outputs by the defuzzifier. The behavior of a fuzzy inference system mainly depends on the constituents of the rules, such as fuzzy sets for antecedent and consequent parts of a rule. Based on these fuzzy sets, different spaces for input and output variables are partitioned. According to this partition, proper functions are created to map input/output spaces to real numbers called membership values.

### Introduction of an adaptive neuro-fuzzy inference system (ANFIS)

A fuzzy inference system can be presented in a neural network form as shown in figure 1b. Such a node-oriented representation is often used for defining a neural network. The intermediate output values of the membership functions and the subsequent logical operations are labeled by circle nodes. The connections are selected in a way that they represent the rule base of the fuzzy system. The basic of fuzzy rules is the binary logic (IF ... AND ... THEN ...). The difference to the binary logic is that the conditions and the results are linguistic variables or terms which reflect fuzzy descriptions of states, so not only 0 or 1 but also values in between.

Based on the network representation, the structure and parameters can be derived from sample data using network-based learning approaches, such as the back-propagation algorithms. A well known neuro-fuzzy system, the adaptive neural fuzzy inference system (ANFIS), was proposed for function approximation [20-23]. It is limited to a special type of FIS proposed by Sugeno [24,25].

In our study, we used the practical neuro-fuzzy system NEFPROX [9-12] which was developed for the model selection of different types of FIS's with hybrid learning algorithms. The algorithm addresses the learning ability of the structure, in which the properties of fuzzy rules are determined. The learning step of these properties is based on the distribution between input and output variables from training data sets. NEFPROX analyses every input-output relation and if no rule already existing in the FIS reflects this behavior, the system creates a new rule which is described by Nauck et al. [11]. The array of input-output relationships created by looking at sequential repetitions of the key element of the decision process to be modelled is then called a "pattern" and from this pattern, fuzzy rules are created In detail the learning algorithm of NEFPROX took one line of the pattern of the training set and searched for each input unit the corresponding membership function. If no rule was found which contained the specific input value and a compatible membership function, NEFPROX creates a new rule node and connected it to the output nodes. For each of these connections NEFPROX searched for a suitable fuzzy weight. This rule creation process was continued until all patterns were analyzed. When, as a practical example, applied to treatment planning, the fuzzy system takes the constraints (which effectively are desired dose points on a certain DVH) as input vectors, then records the resulting respective dose points after one treatment planning iteration as output vectors. Then several planning iterations are performed until a plan that is satisfactory to the planner is achieved. The fuzzy system then takes all the relationships between in- and output vectors over this iterative process (the signature "pattern" of relationships) and creates a set of fuzzy rules to reflect this relationship.

### Experiment design

We divided the experiments into three parts. First we tested the general learning behavior of ANFIS by comparing ANFIS (trained by oFIS) with oFIS based on an arbitrary sets of input vectors resulting in respective output vectors (not a clinical dataset). Then we compared a clinical prostate case planned using oFIS and ANFIS (trained by oFIS). And finally we compared multiple treatment cases (prostate, head and neck ...) planned with oFIS, ANFIS (trained by human knowledge) and human planners.

### Training of ANFIS with the original FIS (oFIS), analysis of the "response" of ANFIS rules as a consequence of changes in oFIS rules

The open source software NFIDENT was used to implement the hybrid learning approach NEFPROX for this study. In a first non-clinical analysis, the ANFIS model was created by the software based on training data. This training data were generated by an existing FIS (oFIS) with known model parameters which were specified manually/directly by a human expert. This approach provides the opportunity to directly assess the process of automatic rule generation in the ANFIS model by comparing the randomly generated sample data consist of input and output vectors, which represent the input-output relationship of oFIS. The input/output data space was uniformly sampled. The data samples were divided into three data sets for model training, validation, and testing purposes. The generalization capability of the new ANFIS was properly controlled by the validation data set. The performance of the model was examined by the testing data set.

Two different analyses proved the ability of NFIDENT to learn the behaviour of the oFIS based on the training/validation data sets. The first analysis addresses the learning efficiency of the rules and the membership functions from the original FIS. The oFIS was edited by using a variable number of rules and was then compared to the resulting ANFIS (Table 1). To analyze the ANFIS' ability to learn membership functions, we changed the behavior of the oFIS by changing numbers in the membership functions and compared the resulting ANFIS (table 2 and 3). To quantify the training error, the mean percentual difference between output vectors of original (manually created FIS) and trained FIS (ANFIS) for a given (identical) set of input vectors was recorded, thus providing an estimate of the "similarity" of the behaviour of the manually created oFIS and the new FIS (ANFIS) trained by the original FIS.

**Table 1:** The results of experimental test in investigating capability of NEFPROX in learning structure of a FIS

**Test No.**	**S**_**E**_	**S**_**N**_	**N**_**Exist**_	**N**_**Partial**_	**N**_**New**_	**Error**
1	8	8	7	1	0	4.2%
2	7	8	7	1	0	3.4%
3	6	8	5	1	2	6.6%
4	5	8	4	1	3	5.7%
5	4	8	2	1	5	4.4%
6	8	8	7	1	0	6.8%
7	8	7	7	0	0	6.6%
8	8	6	6	0	0	10.2%
9	8	5	5	0	0	10.5%
10	8	4	4	0	0	10.1%
11	8	3	3	0	0	11.6%
12	8	2	2	0	0	10.8%
13	8	1	1	0	0	10.1%

Mean						7.77 ± 0.02%

S_E_: The size of the rules used in the existing FIS.

S_N_: The size of the rules used in the new FIS.

N_Exist_: The number of the existing rules in the new FIS and the existing FIS.

N_Partial_: The number of the partially-existing rules in the new FIS and the existing FIS.

N_New_: The number of the non-existing rules in the new FIS and the existing FIS.

Error: Percentual difference between output vectors of original (manually created FIS) and trained FIS (ANFIS) for a given (identical) set of input vectors, thus providing an estimate of the "similarity" of the behaviour of the manually created oFIS and the new FIS (ANFIS) trained by the original FIS

**Table 2:** The results of investigating capability of NEFPROX in learning parameter of membership function (MF) of a FIS focusing on the original oFIS

		**Locations of MF [-1;1.2]**			
	**Membership functions**	**Old FIS**	**New FIS**	**Location Difference**	**Mean Percentage Difference**
Input	MF 1	-1.0	-1.0	0.0	0.83%
	MF 2	1.0	0.9	0.1	
	MF 1	-1.0	-1.0	0.0	
	MF 2	1.0	1.0	0.0	
	MF 1	-1.0	-1.0	0.0	
	MF 2	1.0	1.0	0.0	

Output	MF 1	-1.0	-1.1	0.1	5.0%
	MF 2	0.0	0.0	0.0	
	MF 3	1.0	1.2	0.2	
	MF 1	-1.0	-1.1	0.1	
	MF 2	0.0	0.0	0.0	
	MF 3	1.0	1.1	0.1	
	MF 1	-1.0	-1.1	0.1	
	MF 2	0.0	0.2	0.2	
	MF 3	1.0	1.1	0.1	

**Table 3:** The results of investigating capability of NEFPROX in learning parameter of membership function (MF) of a FIS reflecting the ability of ANFIS to learn differences (changes of the membership function output values -- bold/underlined)

		**Locations of MF [-1;1.2]**			
	**Membership Functions**	**Old FIS**	**New FIS**	**Location Difference**	**Mean Percentage Difference**
Input	MF 1	-1.0	-1.0	0.0	0.83%
	MF 2	1.0	0.9	0.1	
	MF 1	-1.0	-1.0	0.0	
	MF 2	1.0	1.0	0.0	
	MF 1	-1.0	-1.0	0.0	
	MF 2	1.0	1.0	0.0	

Output	MF 1	-1.0	-1.2	0.2	8.88%
	MF 2	**0.5**	0.2	0.3	
	MF 3	1.0	1.2	0.0	
	MF 1	-1.0	-1.2	0.2	
	MF 2	**0.5**	0.4	0.1	
	MF 3	1.0	1.2	0.2	
	MF 1	-1.0	-1.2	0.2	
	MF 2	**0.5**	0.3	0.2	
	MF 3	1.0	1.2	0.2	

### Performance of ANFIS (trained by oFIS) on clinical cases

**Figure 2:**
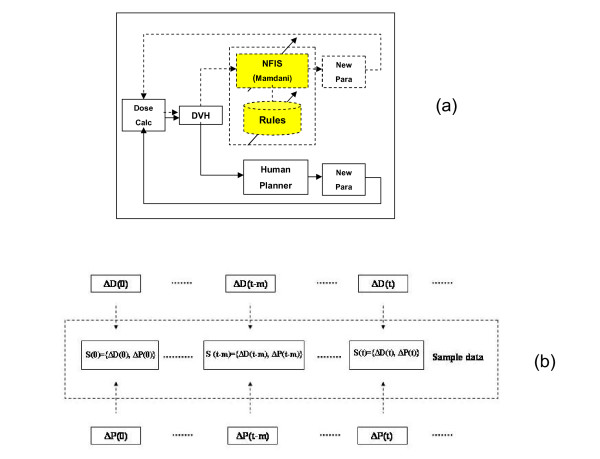
**(a) The AI-guided inverse planning procedure versus the routine procedure as work flow diagram and (b) the resulting sampling data set S(t) from ANFIS**.

To verify the clinical performance of the ANFIS (trained by the oFIS) by using NEFPROX, a treatment plan was generated for a prostate case by the AI-guided inverse planning system. The dose-volume constraint optimization process was fully performed by the ANFIS. For comparison, two treatment plans were generated manually and by the oFIS. The Eclipse^© ^treatment planning system (Varian Medical Systems) provides an application program interface (API) enabling communication between the FIS and the Eclipse^© ^dose calculation and optimization engine. A FIS based program was developed to interactively adjust the parameters (dose-volume constraints and related priorities of a structure) of the objective function after each iteration of dose calculation and plan optimization of the Eclipse^© ^inverse planning system. The workflow of the AI-guided inverse planning procedure versus the routine procedure is shown in figure 2a. The solid line represents the routine procedure conducted by a human planner and the dotted line represents the procedure automatically accomplished by the oFIS built by a human planner. The parameter optimization starts from an initial set of values of the objective function. When a plan was achieved and the dose volume histogram was evaluated, the values of these parameters were modified by either human planner or the FIS programs. The modification procedure continued until a plan with the acceptable dose volume histogram (DVH) was achieved. The resulting mean DVH differences and the standard deviation for the PTV, the bladder, the rectum and the total body structure for 'ANFIS vs. oFIS' and 'ANFIS vs. human planer' are displayed in table 4 and a quantitative analysis of the relevant DVH-parameters was performed. For 11 different characteristic points in the DVH (sampling points at every 10% of maximum dose) the differences of the volumes encompassed by the respective dose volumes between the results for ANFIS vs. FIS and for ANFIS vs. human planner were recorded. To provide a single metric, the mean of these differences was calculated.

**Table 4:** Mean relative volume difference for discrete points selected from the DVH's for prostate for characteristic percentages of isodoses.

	**ANFIS (vs FIS)**	**ANFIS (vs Human)**
PTV	0.822 ± 2.52%	0.774 ± 2.183%
Bladder	18.51 ± 14.24%	14.06 ± 10.83%
Rectum	12.60 ± 18.08%	11.8 ± 17.016%
Body	1.196 ± 1.03%	0.906 ± 0.939%

### Performance of different techniques (manual planning, oFIS, ANFIS trained with clinical constraint data chosen by human planner) on clinical cases

Four clinical cases with typical tumor paradigms (prostate, head and neck, spinal cord, and brain) were tested. The same IMRT field setup, energies, spatial resolutions, dose calculation algorithm (Pencil Beam) and IMRT optimization algorithm were used for each technique when the parameter modification was performed by automated and manual methods respectively. Each case was processed in three different ways (manual method, conventional oFIS method and ANFIS method). The interaction of the FIS-methods with the TPS was provided by an interface which could read out the FIS information and pass it over to the TPS. The interface which connected Eclipse and oFIS/ANFIS is working as follows: In each optimization iteration the optimization module exported all dose-related parameters (mean target volume, mean critical organ and mean tissue) to an interface function which called the designed oFIS/ANFIS. Within this interface function, a new set of dose-volume constraints according to the given dose output was specified. The interface function was called in each optimization iteration and the modified dose-volume constraints took effect in the next iteration. This process continued until the predefined convergence conditions were reached, e.g. the error between calculated and prescribed dose was less than a given number. The fuzzy system therefore exchanges data with the TPS only on the same level that a human planner uses, e.g. providing DVH dose points (constraints) *to *the TPS and taking resulting DVH dose points after an optimization process *from *the TPS [7]. Each method controlled the same default dose-volume constraints so that the control of the methods over the dose distributions was similar.

For this part of the study, ANFIS received human experience as training data. Two human planners (1 dosimetrist and 2 physicists, both experienced in IMRT planning with Eclipse) were observed during their planning process of IMRT with Eclipse. These individuals did not have and did not need to have any expertise with the AI-system because there was no interaction between them and the AI system. In total we observed the planning of 22 clinical patients (mainly prostate cancer) with a median of 7 plan iterations per individual patient until a satisfactory result was obtained. All exchanged parameters of the dose distributions were identical. Specifically, the number of prescription dose points/constraints provided to the TPS was the same for the human planners and the FISs, and all resulting dose points provided by the TPS were used by the FISs. Initially, the TPS calculates based on prescriptions for the structures a dose distribution (DD) and the appropriate DVH which was recorded by screenshot. The human planners evaluate these propositions and optimize the prescriptions by editing DVH dose points for PTV and OAR's. Then the TPS reacts on-the-fly according to these changes, the planner decides if the new DVH corresponds to his expectations and a screenshot is taken. This is done until the planner is confident with the DVH and the captured screenshots reflect the planner work flow. The relevant information such as actual dose DVH(t), prescribed dose CON(t) and weighting factor for every structure in 10% volume step size were readout and stored in a data base. To generate useable data files for the NFIDENT application we used a MATLAB routine to calculate e.g. the relative differences between the constraints and calculated doses and generate a sampling data set S(t) as shown in figure 2b. ΔD(t) describes the difference between the actual plan dose and constraint dose as shown below:

(1)

To train a FIS, the sample data set S(t) had to consist of input and output variables. The input part of training data is ΔD(t) and the output part of training data is ΔP(t) which is calculated as below:

(2)

The resulting sample data sets S(t), one file for every OAR, consist of 6 final vectors, 3 for input (PTV, OAR and normal tissue NT) and 3 for output (PTV, OAR and NT). These sample data sets were processed by the NEFPROX algorithm of the NFIDENT application (chapter 2.2) in order to create fuzzy rules based on the human knowledge described by S(t). These fuzzy rules were exported to the TPS (sample size: pattern with 460 input-output relations) and a previously developed interface provided the possibility for the TPS to use these rules to optimize the treatment plans by editing the prescriptions in the IMRT optimization step.

To quantify the performance of ANFIS vs. oFIS and human planner (the physicist who also provided part of the training plan dataset), for three different characteristic points in the DVH (Volume of respective OAR or target encompassed by 95% of the prescription dose, volume encompassed by 90% of PD and volume encompassed by 50% of PD) the differences for these encompassed volumes between the results for ANFIS vs. FIS and for ANFIS vs. human planner were recorded. To provide a single metric, the mean of these three differences was also calculated, with the results being displayed in table 5.

**Table 5:** Percentage of prescription dose (PD) and percentage of volume (PV) and the mean volume differences for ANFIS, manual planner and FIS

**Sites**	**Anatomic structures**		**Dose**_**ANFIS-MANUAL**_			**Dose**_**ANFIS-oFIS**_			**ΔVolume**_**ANFIS-MANUAL**_	**ΔVolume**_**ANFIS-oFIS**_
			**95% PD**	**90% PD**	**50% PD**	**95% PD**	**90% PD**	**50% PD**	**(Mean)**	**(Mean)**
Prostate	PTV	[% Vol]	-5	-1	0	-8	-3	0	-2	-4
	Bladder	[% Vol]	-5	-15	-34	3	1	4	-18	3
	Rectum	[% Vol]	-13	-16	-18	0	-16	-1	-16	-6
	Body	[% Vol]	0	0	0	0	0	0	0	0

Head & Neck	PTV	[% Vol]	11	0	0	10	-1	0	4	3
	Spinal cord	[% Vol]	0	0	1	0	0	1	0	0
	Lt parotid	[% Vol]	-22	-25	-37	-8	-9	-19	-28	-12
	Rt parotid	[% Vol]	-10	-20	-39	-6	-9	-39	-23	-18
	Body	[% Vol]	0	0	0	0	0	0	0	0

Spinal cord	PTV	[% Vol]	1	-2	0	-4	-3	0	0	-2
	Spinal cord	[% Vol]	-9	-10	-9	-1	-1	-3	-9	-2
	Lt kidney	[% Vol]	0	0	-1	0	0	-1	0	0
	Rt kidney	[% Vol]	0	0	0	0	0	0	0	0
	Body	[% Vol]	0	0	0	0	0	0	0	0

Brain	PTV	[% Vol]	6	3	0	-7	-5	0	3	-4
	Brain stem	[% Vol]	-2	-2	1	-1	-1	-1	-1	-1
	Lt cavernous	[% Vol]	-13	-14	-18	-1	-1	-18	-15	-7
	Optic nerve	[% Vol]	-1	-1	1	-1	0	-1	0	-1
	Body	[% Vol]	0	0	-2	0	0	0	-1	0

## Results

### Training of ANFIS with the original FIS (oFIS), analysis of the "response" of ANFIS rules as a consequence of changes in oFIS rules

The discrepancies between the rules of the ANFIS derived from training by the oFIS and the original rules in the oFIS are summarized in table 1. The numbers of the similar rules, the partially-similar rules and the non-similar rules of both FIS are listed in columns 4-6, respectively. The percentual differences of the output values of the corresponding FIS's are listed in the last column. The results show that the numbers of new rules is increasing as the size of rule base of the oFIS decreased. With a mean value of 7.77 ± 0.02% (percentage of differences for the numerical values) for the output vectors the training error (complete correct learning (oFIS and ANFIS are equal) would have an error of 0%) was low in all tests that show the capability of ANFIS to assume the oFIS behaviour. To compute these percentual errors, we used a set of training data (input/output) to train ANFIS and then used the same training data (only input) to compare the output of the training data with the output results of ANFIS.

The location differences of membership functions in the ANFIS and the oFIS are summarized in table 2 and 3. In these tables, the locations of membership functions for all input and output variables of the original and new FIS's are listed. The last column reports the percentage difference of both FIS's. The mean difference for the input values is 0.83% and for the output values 5.0 to 8.88%.

### Performance of ANFIS (trained by oFIS) on initial clinical case

The resulting treatment plan computed by the ANFIS was compared with the ones achieved by the oFIS and by the manual approach.

**Figure 3:**
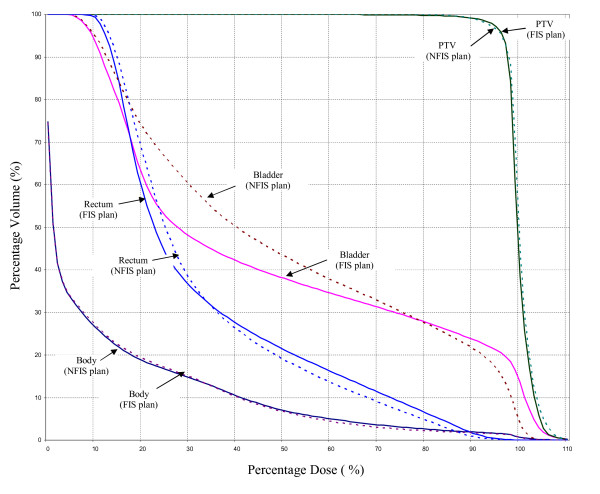
**DVH Comparison of ANFIS and oFIS for a prostate case**.

**Figure 4:**
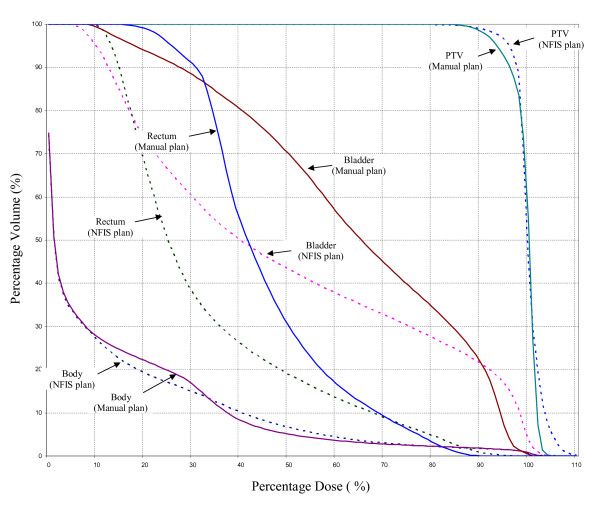
**DVH Comparison of ANFIS and manual planning for a prostate case**.

The DVH comparison of the ANFIS and oFIS in figure 3 showed that comparable dose coverage of the PTV was achieved. There are minor differences regarding the dose to bladder and rectum while the integral dose to the whole body structure was nearly identical. Table 4 shows that the mean difference for the PTV volume values for characteristic percentages of isodoses between ANFIS and oFIS is 0.822 ± 2.52%. The same data is provided for OAR's. The DVH comparison in figure 4 showed that the dose distribution generated by the ANFIS actually outperformed the one achieved by manual planning (trial-and-error method). The mean PTV volume difference was 0.774 ± 2.183% for the ANFIS approach and the human plan as reported in table 4.

### ANFIS (trained by human knowledge) on multiple clinical cases

Synoptically, comparing multiple clinical cases between ANFIS and human planner, comparable PTV coverage was achieved, while the OAR volumes encompassed by various isodoses were typically smaller for ANFIS-generated plans (by an average of 7.4%). Comparing ANFIS and oFIS for the same cases, PTV coverage was somewhat inferior for ANFIS generated plans, although this was a minor difference (mean reduction of volumes encompassed by a set of characteristic isodoses was only 1.5%). For OAR volumes encompassed by characteristic percentages of isodoses, a mean reduction between 0 and 28% was recorded. These results are calculated based on raw data displayed in table 5.

### Head and Neck

**Figure 5:**
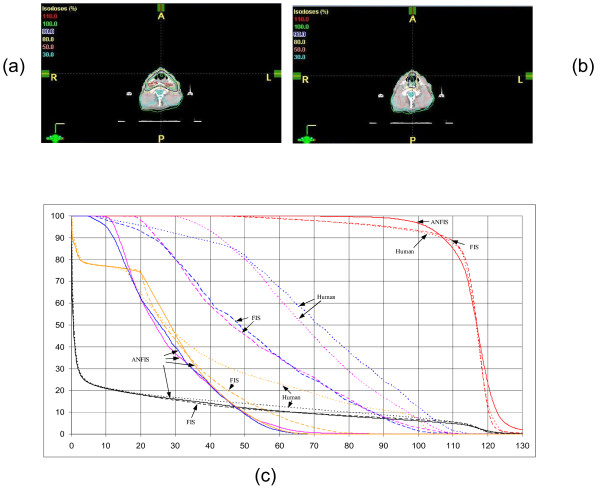
**Head and neck plan evaluation for (a) ANFIS and (b) human plan**. The DVHs are shown in (c). ANFIS is displayed as solid lines, plans created by human as dot lines and FIS as dashed lines. The red lines represent the PTV, the blue/pink lines the right/left parotid, the orange lines the spinal cord and the black lines the normal tissue.

Nine coplanar equal-spaced beams were used in this case. The dose-volume histograms and dose distributions for all anatomical structures were plotted in figure 5. We observed improvements of the dose coverage on a majority of the PTV in the plan generated by the ANFIS method compared with the oFIS and the manual plan. As a trade-off, a small area of PTV received higher doses. The mean dose to left and right parotids in the ANFIS plan were 20% lower than the ones achieved by the oFIS plan and 40% lower than the manual plan. The spinal cord is exposed to the lowest dose in both FIS plans. The maximal dose to the spinal cord is 34% lower for ANFIS than the one achieved by the manual planner. There is no visible change of normal tissue dose in the three plans. A summarized overview of the differences of discrete DVH difference points is shown in table 5.

### Prostate

**Figure 6:**
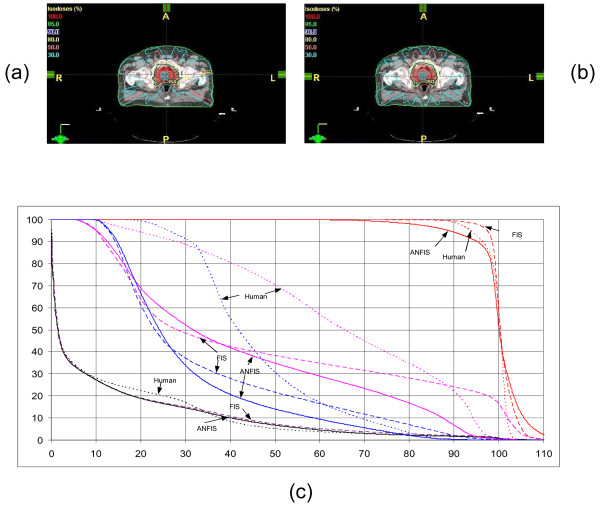
**Prostate plan evaluation for (a) ANFIS and (b) human plan**. The DVHs are shown in (c). ANFIS is displayed as solid lines, plans created by human as dot lines and FIS as dashed lines. The red lines represent the PTV, the blue lines the rectum, the pink lines the bladder and the black lines the normal tissue.

The anatomic structures on the central slice of the treatment planning CT and beam configuration are demonstrated in figure 6a/b. Nine coplanar equally-spaced beams were used. As displayed in figure 6c, the dose to a certain characteristic percentage volume of the plan achieved by ANFIS was compared with those of plans achieved by a human planner and the oFIS and their differences are summarized in figure 6c. PTV coverage provided by ANFIS is not optimal compared to the manual plan and oFIS plan. The prostate plan generated by ANFIS showed inferior dose coverage (as indicated by the lower value for the dose encompassing 90% of the PTV) and larger areas with exposure to higher doses (hot spots). As a trade-off, the doses to critical organs are significantly improved. We observed about 20% dose reduction to 50% of the volumes for rectum and bladder and 10% dose reduction to 20% of the volumes for rectum and bladder.

### Brain

**Figure 7:**
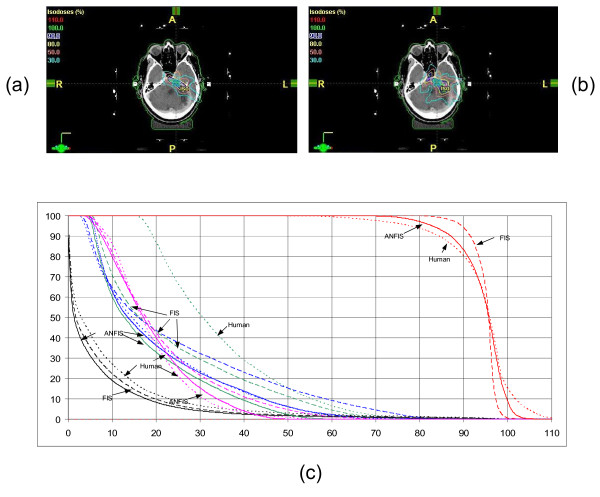
**Brain plan evaluation for (a) ANFIS and (b) human plan**. The DVHs are shown in (c). ANFIS is displayed as solid lines, plans created by human as dot lines and FIS as dashed lines. The red lines presents the PTV, the blue lines the brain stem, the pink lines the optic stem, the green lines the left cavar and the black lines the normal tissue.

Ten non-coplanar beams were used in this case. As displayed in figure 7, the dose coverage on a majority of the PTV achieved by the ANFIS method was improved compared to the manual plan, but slightly worse than the oFIS plan. For the dose to brain stem, oFIS and ANFIS showed improvements to the manual plan. Comparing the dose to the left cavernous sinus, the ANFIS plan was better than the manual plan, also an improvement to the oFIS plan (4% lower median dose to the structure in the ANFIS plan vs. the oFIS plan) was observed. For the optic nerve, the maximal dose recorded in the ANFIS-plan was similar to the one achieved by the manual plan and 30% less than the one achieved by the oFIS plan.

### Spinal cord

**Figure 8:**
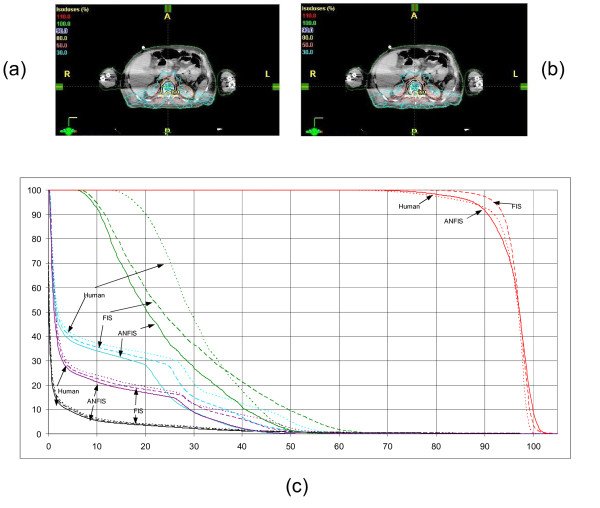
**Spinal cord plan evaluation for (a) ANFIS and (b) human plan**. The DVHs are shown in (c). ANFIS is displayed as solid lines, plans created by human as dot lines and FIS as dashed lines. The red lines presents the PTV, the turquoises/purple lines the left/right kidney, the green lines the cord and the black lines the normal tissue.

Seven coplanar beams all coming from the posterior body half were used in this case. As displayed in figure 8, the dose coverage on the majority of the PTV in the plans generated with ANFIS was improved compared to the manual plan, but worse in comparison to the oFIS plan. The cord dose was improved (4% lower median dose to the structure in the ANFIS plan vs. the oFIS plan and manual plan). The left kidney dose for 40% volume is 6% and 10% less than those doses in oFIS and manual plan. The right kidney dose is less changed in ANFIS.

## Discussion

Multiple optimization strategies for IMRT have been investigated in order to achieve the best possible plan results. A perfect TPS would run the treatment planning process without relevant human interaction based on an optimization process driven by biological models for tumor control- and side effect probability. While this line of thought of displaying objective arguments to the planner, thus facilitating the planner decision is already being followed [26] and is the most appealing approach, there is still uncertainty regarding the underlying model parameters. With increasing availability of solid data regarding these parameters this approach will gain more and more importance, but for the time being, planners still mainly operate constraint based TPSs using rather fuzzy individual physician's biological "ideas". A system such as the one introduced by us therefore still has the potential to shorten this process, facilitating relating the individual planner's biological "idea" to a constraint based TPS. Although the system was established in conjunction with a specific TPS, its principle (providing a set of constraints to the interface where usually human planners input their constraints/preferences) can be used with all constraint driven inverse TPS. The system interacts with the TPS only on a level where the normal interaction of the human planner with the TPS takes place. By definition the tool therefore drives the entire planning system in a way that is identical to what the human planner does. Although interacting with the TPS at the level of deliverable plans would probably yield better plans, the approach could then not that easily be generally applied to a wide range of constraint driven TPS.

Other strategies also operate on such a meta-level. An example is the multi-objective plan database for multi-criteria optimization (MCO) [27]. Craft et. al. found that in order to do effective MCO for IMRT, it was not necessary to build a large number of plans, which reduces the overall planning time. While this approach offers a more systematic method to find a suitable plan than sequential optimization with arbitrarily changed constraints, a downside is that the planner has to analyse every plan and has to decide subjectively which plan is the best based on the DD.

Another approach that has already been followed for some time is based on Pareto fronts (set of Pareto optimal solutions, with Pareto optimal meaning that it is not possible to improve one objective without deteriorating at least one of the others) [28]. Spalke et al. investigated also in this area of multiobjective radiotherapy planning. They proposed two methods to analyse the database systematically (principal component analysis and isomap method) which were able to extract key trade-offs and provide information for a better understanding of IMRT planning [29].

Our study explored another option, showing the ability to use a fuzzy system to automate the plan optimization process at the input level of a constraint based system. The evaluated ANFIS system comprehends subjective knowledge of 2 planners, thus offering the possibility to integrate the knowledge of an infinite number of planners to continuously improve its performance. The chosen data amount of 22 clinical cases with a median of 7 plan iterations per case led to a sufficient amount of training data for the ANFIS but further data acquisition from more human planner should be done to extend the different datasets. While "mimicking" planner behavior is a "phenomenological" approach, it nevertheless offers the possibility to quickly establish an automated planning process that reliably delivers results in accordance with human planners' objectives. We could also show that a FIS can be built as an adaptive-neuro FIS from training samples using a neuro-fuzzy function approximation system.

An important issue in this study was the evidence of the FIS's capability to learn the input-output relationship of a known or unknown AI system provided a large number of rules are used. Investigations, in other fields than radio oncology confirmed this statement [30,31]. Users might expect that the major properties of the ANFIS were close to those of the oFIS. However, this is not necessarily the case since these rules were automatically learned from data in order to minimize the difference between the outputs of network-based FIS and oFIS. The usage of larger data bases by importing more human planners into the training data base will improve the ANFIS accuracy [32]. The output difference decreased with an increasing number of rules. As shown in table 1, when the number of rules in the ANFIS was chosen equally to that of the oFIS (e.g. 8 rules), the least discrepancy was observed as well as the learned rules of the ANFIS almost always belong to the oFIS. It is noted that when the number of the rules is reduced, the approximation capability will be compromised. Furthermore we showed in table 2 and table 3 the ability of ANFIS to learn exact membership functions from an existing system in a proper manner.

The resulting dose distributions generated by such an ANFIS were nearly identical to those achieved by the oFIS and slightly better than by an expert human planner. Since the structure and parameters of the ANFIS were learned from sample data by a learning algorithm, they might not be identical to those of the oFIS but an amplification of the learning data base may lead to advantages over oFIS.

Observing the clinical adaptability, we showed in multiple trials with training data from oFIS and from human planner the excellent performance of the trained ANFIS. For characteristic percentages of isodoses a mean volume reduction for organs at risk (OAR) of 7.4% for ANFIS vs. human planner and 3% for ANFIS vs. oFIS was achieved. For PTV coverage an improvement of 1.5% for ANFIS vs. human planner and a reduction of -1.75% for ANFIS vs. oFIS was observed.

Several factors may contribute to the observed heterogeneity between plans with oFIS, ANFIS and the human planner that is not immediately intuitive. The treatment plan was not parameterized exhaustively. As it can be seen in the sample prostate plan, there is considerable variation between plans with regard to femoral neck dose, which is not explicitly processed during the planning procedure by the fuzzy system. The human planner, however, may still implicitly record and process this OAR.

Another issue that explains the sometimes significant differences between the approaches is the fact that the training data was recorded across three different planners while the plans derived from a human planner were all created by only one of these three individuals. Heterogeneity between the result from human planning (one planner) and from automatic planning (knowledge based on three different planners) might be a consequence of very different "ideas" of what constitutes an optimal plan for these three different individuals.

Finally, the training was mainly performed on prostate paradigms and then applied to prostate but also to very different entities. It is possible that the performance on these other paradigms is even less predictable. A future line of research should therefore also evaluate the use of entity-specific sets of fuzzy rules, generated based on homogeneous training data.

The generation to build a FIS from sample data using a neuro-fuzzy system takes only a short time (few seconds) and has to be done only once. As we gain the capability to quickly build a FIS, the next problem is to collect proper and sufficient sample data from clinical practice as described in section "Performance of ANFIS (trained by oFIS) on clinical cases".

As mentioned by Yan et. al. the choice of inference rules is essential for a fuzzy inference system [8]. In the future, a protocol will be developed to collect the sample data from clinical practice of treatment planning, and a hybrid learning approach to combine manual specification and automatic learning of model parameters will be implemented

## Conclusion

A technique using a neuro-fuzzy system for automated model learning of a FIS was successfully established in this study. Multiple clinical cases proved the compatibility of FIS and commercial TPS and the potential to assume the optimization step of IMRT planning without iterative human interaction to reduce the clinical workload of human planners. The neuro-fuzzy system provides an effective way for model selection of FIS from practice data. Based on this technique, future parameter optimization of inverse planning is guided by the prior knowledge directly conveyed from multiple clinical data sets instead of having to manually create rules for such a system. Such a system is therefore able to "learn" directly from a human planner and emulate the respective behavior.

## Competing interests

The authors declare that they have no competing interests.

## Authors' contributions

FS participated in the experiment design, carried out the experimental work of the study and drafted the manuscript. HY conceived the study, supervised the execution and helped to draft the manuscript. FL and FW have been involved in data interpretation and drafting the manuscript. FFY conceived the study and helped drafting the manuscript.
